# Pathways from Exposure to Community Violence to Bullying Victimization among African American Adolescents in Chicago’s Southside

**DOI:** 10.3390/ijerph19159453

**Published:** 2022-08-02

**Authors:** Jeoung Min Lee, Jun Sung Hong, Stella M. Resko, A. Antonio Gonzalez-Prendes, Dexter R. Voisin

**Affiliations:** 1 School of Social Work, Wichita State University, Wichita, KS 67260, USA; 2 School of Social Work, Wayne State University, Detroit, MI 48202, USA; eg2092@wayne.edu (S.M.R.); aa3232@wayne.edu (A.A.G.-P.); 3 Jack, Joseph and Morton Mandel School of Applied Social Sciences, Case Western Reserve University, Cleveland, OH 44106, USA; drv22@case.edu

**Keywords:** adolescents, bullying victimization, depression, exposure to community violence, poverty

## Abstract

The present study proposes and examines the pathways from exposure to community violence to bullying victimization through the influences of depression, exposure to peer delinquency, and drug use among 638 African American adolescents (aged 12–22) from low-resourced neighborhoods in Chicago’s Southside. The study found that African American adolescents who were exposed to community violence were likely at risk of bullying victimization, depression, exposure to peer delinquency, and drug use. Depression can heighten the risk of bullying victimization. These findings have implications for future research.

## 1. Introduction

The rate of exposure to community violence is significantly higher among urban African American children and adolescents than among children of other racial and ethnic groups [1,2]. *Community violence* is defined as violence that occurs in a neighborhood, such as homicide, shootings, assault, robberies with assault, and rape [3]. According to the National Survey of Children’s Exposure to Violence, 46% of youths, in general, reported being physically assaulted, and 19% reported witnessing an assault in their community [4]. Of these youth, 12.8% were African Americans with the highest rate of exposure to multiple forms of victimization [4].

Community violence has been frequently reported in Chicago’s Southside, which has long experienced economic inequality, leading to residential segregation [5]. Youth in Chicago’s Southside, African Americans in particular, have experienced or witnessed institutional discrimination, limited job opportunities, poor infrastructure, racial profiling, violent crimes, and social isolation [6]. Relative to Whites in metropolitan areas, urban African Americans have a significantly higher rate of residing in impoverished communities [7]. Structural barriers are likely to contribute to feelings of being discriminated against, oppressed, and marginalized, and adapting to these structural disadvantages can negatively impact one’s behaviors and relationships with others. Scholarships on exposure to community violence have reported that exposure to community violence contributes to lower academic performance, drug use, antisocial behavior, school disengagement, negative relationships, psychological distress, and maladjustment, as many studies have shown [1,8,9,10,11,12,13,14].

An emerging body of research has also explored how exposure to community violence might predict bullying. Over the years, bullying and aggressive behaviors have been frequently examined as outcomes of exposure to community violence [10,15,16,17,18,19,20]. The relationship between exposure to community violence and bullying victimization, however, has been less frequently explored. Given that African Americans in urban neighborhoods are chronically exposed to violence, such as shootings and stabbings [21,22], it is not surprising that these youth are likely to report feeling unsafe in their neighborhood [23] and are frequent targets of bullying and other forms of peer violence. A limited number of research findings suggest that adolescents who witness violence in their community are at an increased risk of bullying victimization.

Adolescents who are chronically exposed to violence in their community rarely become victims of bullying immediately; they likely follow pathways by which bullying victimization is increased when exposed to community violence. The pathways from exposure to community violence to bullying victimization have been supported by various theoretical perspectives. For instance, the general strain theory proposes that certain stressors or strains (e.g., exposure to community violence) are likely to produce negative emotions (e.g., depressive symptoms) and unhealthy coping mechanisms [24], such as affiliation with deviant peers and engaging in substance use. The social disorganization theory, on the other hand, postulates that affiliation with deviant peers and substance use would be attributed to impoverished economic and social conditions (e.g., characterized by community violence) that would limit the community’s ability to supervise or regulate adolescent behavior (e.g., bullying victimization) [25]. Opportunity theory would posit that the likelihood that an adolescent will become a victim of bullying is shaped by the following factors: (1) the presence of a motivated offender (e.g., affiliation with deviant peers), (2) the lack of a capable guardian, and (3) youths’ participation in activities that shape their exposure and proximity to motivated offenders [26,27].

The present study explores pathways from exposure to community violence to bullying victimization through the influences of depression, exposure to peer delinquency, and drug use from a sample of urban African American youth in Chicago’s Southside.

### 1.1. Mechanisms Linking Exposure to Community Violence and Bullying Victimization

Exposure to community violence has been shown to contribute to mental health problems, and studies have documented a significant relationship between exposure to community violence and depressive symptoms in adolescents [28,29]. Youth who are exposed to violence in their community are likely to perceive their surrounding as unsafe or feel unworthy of feeling safe [30], which might contribute to feelings of hopelessness and depression. Depression, in turn, is an antecedent of bullying victimization, as indicated by several study findings [31,32]. According to Hodges et al. [32], internalizing problems (including depressive symptoms) and physical weaknesses were predictors of bullying victimization risks. Isaacs et al. [33] also documented that a cyclical process of internalizing problems and the changes in self-perceived social competence would likely increase bullying victimization. Depressive symptoms could make the adolescent an easy target for bullying victimization because adolescents who are depressed may be perceived as being more vulnerable and an easy target [31].

Exposure to peer delinquency is another possible mechanism that amplifies the association between exposure to community violence and bullying victimization. Among some African American youth in urban areas, exposure to community violence might contribute to weakened conventional norms and compromised prosocial behaviors, and as a result, these youth are likely to turn to delinquent peers [34] who might display aggressive tendencies in order to maintain their status in their peer group [35]. Exposure to peer delinquency can also influence maladaptive behaviors in adolescents, such as substance use [36] and bullying victimization risks. As highlighted by the opportunity theory [27], youth who are consistently exposed to violence might be inclined to seek friendships with non-conventional peers [37] as they perceive befriending “conventional” peers to be a challenge. These adolescents subsequently seek peers who engage in maladaptive behaviors, including bullying, which might put them at risk of bullying victimization.

Some adolescents who are exposed to violence in their community might also be inclined to turn to drugs [8,38,39]. According to one study, which included a sample of African American high school students, being a victim of violence in the neighborhood, witnessing violence in the neighborhood, and urban hassles were associated with an increase in alcohol and marijuana use [39]. Exposure to community violence was found to be correlated with other types of drug use including cigarettes, crack, and hard drugs in another study [38]. Adolescents who are exposed to community violence are also at an elevated risk of negative social influences (i.e., friends using substances), which can increase their psychosocial distress, contributing to substance use [40]. As supported by the self-medication hypothesis, youth who are consistently exposed to community violence likely experience strain and might turn to illicit drugs in an effort to cope with strain [41].

Alcohol and drug use are rarely considered antecedents of bullying victimization in research. However, it is conceivable that adolescents who misuse alcohol and drugs might be vulnerable to bullying victimization as they tend to be perceived as easy targets when they are intoxicated. These adolescents may also be targeted by their “conventional peers” who might perceive them as “troublemakers” for participating in delinquent activities.

### 1.2. Study Hypotheses

Based on the aforementioned research findings and theoretical supports, the following hypotheses are proposed and tested: (a) exposure to community violence will be positively associated with bullying victimization (*direct effect*); (b) exposure to community violence will be positively associated with depressive symptoms, which will be positively associated with exposure to peer delinquency and drug use, thereby increasing the likelihood of bullying victimization (*indirect effects*). 

## 2. Materials and Methods

Data for the study were collected from 638 African American early to late adolescents, ages 12 to 22, in two high schools, one church youth group, two community youth programs, and four public areas (e.g., parks, fast food outlets, malls, and movie theaters) in the Southside of Chicago from August 2013 to January 2014. Among the study samples, 476 (74.6%) received government assistance, 45.5% were male, 54.2% were female, and the mean age was 15.84 (*SD* = 1.41) (see Table 1). The last author received an approval from the Institutional Review Board of the University of Chicago and collected the data. Flyers with information about the study were posted at high schools, churches, youth community centers, and public sites that were frequented by youth. Also, trained research assistants provided information about the study to the potential participants. Once permission was obtained from high school principals and leaders of a church youth group and community youth programs, the research assistants provided consent letters to the interested adolescents and their caregivers. The participants and their parents returned the signed consent letters. Research assistants also recruited adolescents who were with their caregivers at public sites and provided them with a consent letter. The participants completed the survey under the supervision of the research assistants. The survey lasted about 45 min, and the participants were each given $10 once they completed the survey.

### 2.1. Measures

*Bullying victimization* consisted of four items that were adapted from the University of Illinois Victimization Scale (UIVS) [42], including “Other students pick on me,” “Other students made fun of me,” “Other students called me names,” and “I got hit and pushed by other students.” Response options were *Never (0), 1 or 2 times (1), 3 or 4 times (2), 5 or 6 times (3)*, and *7 or more times (4)* during the past 30 days. The internal reliability score for the items in this study was α = 0.87. This scale has been widely used to measure bullying victimization among U.S. adolescents and has good validity and high internal consistency with U.S. samples. Alpha ranged from 0.84 to 0.90 in these studies [43,44,45].

*Depression* was measured with five items, which were adapted from the Harvard National Depression Screening Scale (HANDS) [46], and includes “Feeling no interest in things during the past 7 days,” “Feeling blue,” “Feeling hopeless about the future,” “Feeling so restless you couldn’t sit still,” and “Thoughts of ending your life.” Response options were *Not at all (0), A little bit (1), Moderately (2), Quite a bit (3),* and *Extremely (4).* The internal reliability score for the items was *α* = 0.77.

*Exposure to community violence* consisted of seven items from the Exposure to Violence Probe [47], which included the statement, “During your lifetime, how often have the following events occurred:” and was followed by “Has a close relative or friend been robbed or attacked?”, “Has a close relative or friend been seriously injured because of violence?”, “Has a close relative or friend been robbed or attacked?”, “Have you seen a dead body, not in a funeral?”, “Have you seen someone being beaten?”, “Have you been a victim of violence?”, and “Have you witnessed a gun-related incident?” Response options were *0 times (0)* to *6 times (6)*. The internal reliability was *α* = 0.87.

*Exposure to peer delinquency* was comprised of nine items from the Adolescent Delinquency Questionnaire [48], which included “How many of your ten closest friends drink alcohol?”, “How many of your ten closest friends skip school or class?”, “How many of your ten closest friends have smoked marijuana?”, “How many of your ten closest friends have used drugs?”, “How many of your ten closest friends smoke cigarettes?”, “How many of your ten closest friends have smoked marijuana?”, “How many of your ten closest friends carry guns?”, “How many of your ten closest friends use weapons?”, and “How many of your ten closest friends have stolen something worth more than $5?” Response options were *None (0), A few (1), About half (2), Many (3)*, and *Most (4)*. The internal reliability scores reported in previous studies ranged from 0.86 to 0.89 [49,50].

*Drug use* was measured using five items, beginning with the statement, “During the past 30 days, on how many days did you” followed by “Smoke cigarettes or cigarillos”, “Use Ecstasy (Molly, MDMA)”, “use Lean or Krokodil (cough syrup, codeine)”, “use marijuana (blunts, pot, weed)”, and “Use the crack and/or cocaine” Response options were *0 days (0), 1 day (1), 3–5 days (2), 6–9 days (3), 10–19 days (4), 20–29 days (5),* and *All 30 days (6)*. The internal reliability score for the items was *α* = 0.70.

The covariates include *biological sex* (Male [1], Female [2]), *age* (fill-in-the-blank), and *government assistance* (“Are you receiving free or reduced lunch and/or Supplemental Nutrition Assistance Program (SNAP) benefits?”; No [0], Yes [1]).

### 2.2. Analytic Techniques

Analyses for the study included descriptive statistics, Pearson’s coefficient correlations, and structural equation modeling (SEM) with Mplus8.0 [51]. The hypotheses are tested, controlling for biological sex, age, and government assistance. The SEM consisted of (a) direct association (Hypothesis 1) and (b) indirect associations (Hypothesis 2). The multiple indices, including the comparative fit index (CFI), Tucker–Lewis index (TLI), standardized root square mean residual (SRMR), and root mean square error of approximation (RMSEA), were used to assess the model fit [52,53], and the maximum likelihood (ML) and bootstrapping method were used in the SEM to estimate the indirect associations for the specific pathways.

## 3. Results

Table 1 displays the results of the correlation analysis. Exposure to community violence was positively associated with bullying victimization (*r* = 0.37, *p* < 0.001), exposure to peer delinquency (*r* = 0.09, *p* < 0.05), and drug use (*r* = 0.20, *p* < 0.001).

**Figure 1:**
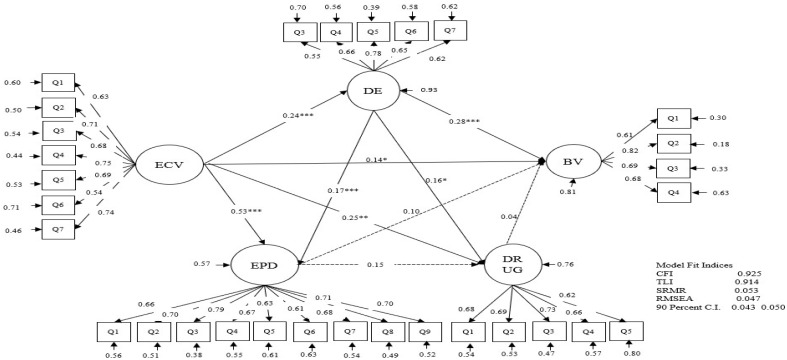
Estimates of the Pathways from Exposure to Community Violence to Bullying Victimization. *Note*. BV = bullying victimization, ECV = Exposure to community violence, EPD = Exposure to peer delinquency, DE = Depression, DRUG = Drug use. The asterisk mark is only displayed for the pathways between the study variables. The effects of the covariates on the variables were omitted in the figure. Please refer to the Measures section for indicators (Q1~Q9) of the latent variables. * *p* < 0.05; ** *p* < 0.01, *** *p* < 0.001.

The goodness-of-fit indices for the path model estimated CFI = 0.925, TLI = 0.914, and RMSEA = 0.053 (90% confidence intervals [CI] = 0.043–0.050, SRMR = 0.047), which indicated that the path model represented an acceptable model fit. Table 2 shows the results of the estimated direct associations and covariances among the study variables, and Figure 1 shows the direct associations among the study variables, which were latent variables.

Exposure to community violence was positively associated with bullying victimization (*β* = 0.140, *p* = 0.036) and depression (*β* = 0.243, *p* = 0.000), exposure to peer delinquency (*β* = 0.530, *p* = 0.000), and drug use (*β* = 0.247, *p* = 0.004). Also, depression was positively associated with exposure to peer delinquency (*β* = 0.171, *p* = 0.000), drug use (*β* = 0.163, *p* = 0.022), and bullying victimization (*β* = 0.278, *p* = 0.000).

The estimated indirect associations between exposure to community violence and bullying victimization through depression, exposure to peer delinquency, and drug use as mediators are shown in Table 3. Exposure to community violence was indirectly and positively associated with bullying victimization (*β* = 0.141, 99% CI = 0.023–0.259). One significant indirect pathway was shown: exposure to community violence → depression → bullying victimization (*β* = 0.058, 99% CI = 0.016–0.120).

Regarding the covariates, age (*β* = 0.166, *p* = 0.000) and government assistance (*β* = 0.083, *p* = 0.037) were positively associated with exposure to community violence, and male sex was negatively associated with exposure to community violence relative to female sex (*β* = −0.172, *p* = 0.000). Age (*β* = −0.204, *p* = 0.000) was negatively associated with bullying victimization, while sex and SES were not significant. Female sex (*β* = 0.147, *p* = 0.001) was positively associated with depression, but age and SES were not significant. Male sex (*β* = −0.124, *p* = 0.001) was positively associated with exposure to peer delinquency, and age (*β* = 0.144, *p* = 0.000) was negatively associated with exposure to peer delinquency, while SES was not significant. Also, male sex (*β* = −0.151, *p* = 0.000) was negatively associated with drug use, but it was not associated with age and SES.

## 4. Discussion

This current study proposed and empirically tested potential pathways from exposure to community violence to bullying victimization among African American adolescents in Chicago’s Southside. More specifically, the study examined whether depression, exposure to delinquent peers, and drug use potentially mediated the association between exposure to community violence and bullying victimization, controlling for biological sex, age, and government assistance. In terms of the direct effects, the findings indicated that exposure to community violence was positively associated with depressive symptoms, which supported our proposed hypothesis and prior research findings [28,29]. Our findings also suggest that exposure to community violence was positively associated with exposure to delinquent peers and drug use. Living in an unsafe neighborhood can be highly stressful for adolescents who consistently are in fear for their safety. These adolescents might seek “non-conventional” peers for support and to protect themselves as they may have difficulty forming friendships with “conventional” peers [34]. They might also seek unhealthy coping mechanisms, such as using drugs [8,54] in an effort to self-medicate.

The indirect effect showed that youth who were exposed to community violence were likely to use drugs, which increased their risk of bullying victimization. Youth, particularly African American youth, who reside in poor inner cities and high-crime areas are more frequently exposed to community violence than their suburban and White peers [10]. Given that Chicago’s Southside has a long history of racial discrimination and structural disadvantages [55,56], which resulted in increased rates of crime and violence, it is not surprising that African American youth in urban neighborhoods are likely to report feeling unsafe from an early age. These adolescents may be more vulnerable to violence than their peers of other racial and ethnic groups due to structural disadvantages. Moreover, being chronically exposed to community violence is a stressful life event, which is likely to contribute to mental health problems (e.g., depression, drug use, externalizing problems) [57,58] and bullying victimization [15]. Although youth learn to adapt to stress in their communities, strains, such as exposure to community violence are likely to increase the risk of bullying victimization.

### Limitations and Implications for Research

Several limitations of this study need to be discussed. This study utilized a cross-sectional research design, so causal inferences cannot be made. A longitudinal research design is needed to estimate the time-order effects to explore developmental pathways from exposure to community violence to bullying victimization. Also, self-reported measures were used, which likely have resulted in self-reporting bias. Future research should include reports from parents, peers, and teachers, which can increase the validity of the findings. Moreover, the sample for this study was derived exclusively from Chicago’s Southside. Because cultural contexts and characteristics vary in different urban areas, it is difficult to generalize these findings to African American adolescents in other urban areas. Future research should include samples from various urban areas. Additionally, the study did not consider other relevant factors that are likely to be indirectly associated with bullying victimization. Future research should consider variables that might be related to exposure to community violence and bullying victimization, such as a lack of resources and social support in the schools and community. More importantly, future research needs to consider how disadvantages, such as racism, discrimination, and lack of policing might be related to adolescents’ risk of exposure to community violence, depression (and other internalizing problems), exposure to delinquent peers, drug use, and bullying victimization. And finally, future research might consider exploring sex and gender differences in the association between exposure to community violence and bullying victimization, as exposure to community violence would differentially impact male and female adolescents’ psychological wellbeing and their relationships with others.

## 5. Conclusions

Findings from the current study highlight the importance of not only examining variables that are directly associated with bullying victimization but also variables that explain how community-level variables might be related to adolescents’ experiences in bullying victimization. In Chicago’s Southside, perceived institutional discrimination, lack of job opportunities, poor infrastructure, or social isolation have resulted in social and economic segregation. Many residents experience discrimination and structural disadvantages on a daily basis, which would undermine their mental and behavioral health, as well as relationships with others. Therefore, researchers need to further explore community-level factors and how they may negatively affect the mental and behavioral health of adolescents. Understanding these community-level factors can facilitate the development of practice and policy that would recognize adolescent bullying not only as an individual-level problem but also as a macro-level problem that is influenced by existing structural disadvantages. When developing a program or policy for youth in low-resourced, urban neighborhoods, policymakers, in collaboration with practitioners and other relevant stakeholders, need to take into consideration these structural disadvantages, which tend to increase adolescents’ risk of depressive symptoms, delinquent peer affiliations, and drug use. Structural disadvantage continues to be a serious concern in low-resourced urban neighborhoods, and poverty, in particular, is a major barrier to help-seeking. Social workers who are tasked to provide services in urban neighborhoods need to work with community leaders in advocating for necessary resources, such as culturally-relevant mental health services for adolescents who are frequently exposed to violence in their community, which could ultimately lead to fewer behavioral problems, such as bullying. 

## Figures and Tables

**Table 1:** Descriptive Statistics and Correlations among the Study Variables.

Variable	*n*	*M*	*SD*	1	2	3	4	5	6	7	8
1. Age (12–22)		15.84	1.41	-							
2. Biological sex (ref. female)				−0.14 ***	-						
Female	346										
Male	290										
3. Government assistance (ref. yes)				0.10 *	0.02	-					
Yes	476										
No	153										
4. Bullying victimization (0–16)		2.13	3.22	−0.02	−0.01	−0.08 *	-				
5. Exposure to community violence (0–42)		10.01	9.19	0.03	0.00	0.01	0.37 ***	-			
6. Depression (0–20)		3.49	3.80	0.00	0.04	0.06	0.15 ***	0.09 *	-		
7. Exposure to peer delinquency (0–36)		8.31	7.56	0.02	0.02	0.06	0.14 **	0.06	0.10 *	-	
8. Drug use (0–18)		1.74	3.26	0.11 **	−0.11 **	−0.02	0.14 ***	0.20 ***	0.07	0.04	-

* *p* < 0.05; ** p < 0.01; *** p < 0.001.

**Table 2:** Estimated Direct Effects and Covariances.

	Estimate	SE	CR	*p*-Value		Estimate	SE	CR	*p*-Value
Regression Weight					Regression Weight				
BV←ECV	0.140	0.067	20.093	0.036	DRUG←ECV	0.247	0.086	2.889	0.004
←DE	0.278	0.058	4.791	0.000	←EPD	0.154	0.113	1.359	0.174
←EPD	0.100	0.070	1.425	0.154	←DE	0.163	0.071	2.298	0.022
←DRUG	0.043	0.060	0.716	0.474	←Sex	−0.151	0.043	−3.527	0.000
←Sex	0.011	0.041	0.271	0.787	←Age	0.060	0.045	1.326	0.185
←Age	−0.204	0.038	−5.337	0.000	←SES	−0.031	0.042	−0.737	0.461
←SES	−0.026	0.037	−0.707	0.480					
DE←ECV	0.243	0.050	4.901	0.000	Covariances				
←Sex	0.147	0.043	3.403	0.001	Sex <-> ECV	−0.172	0.044	−3.914	0.000
←Age	−0.011	0.050	−0.212	0.832	Age <-> ECV	0.166	0.042	3.904	0.000
←SES	0.004	0.040	0.094	0.925	SES <-> ECV	0.083	0.040	20.080	0.037
EPD←ECV	0.530	0.046	11.566	0.000					
←DE	0.171	0.047	3.660	0.000					
←Sex	−0.124	0.038	−3.268	0.001					
←Age	0.144	0.038	3.78	0.000					
←SES	−0.017	0.035	−0.475	0.635					

*Note.* SE = Standard error, CR = Critical ratio, BV = Bully victimization, ECV = Exposure to community violence, EPD = Exposure to peer delinquency, DE = Depression, DRUG = Drug use. Reference variables are: Sex = female and SES (government assistance) = yes.

**Table 3:** Direct and Specific Indirect Associations between Exposure to Community Violence and Bullying Victimization.

	Direct	Indirect								
		→EPD	→DRU	→DE	→DE	→EPD	→DE	→DE	Total Indirect	Total
					→EPD	→DRUG	→DRUG	→EPD		
								→DRUG		
Estimates	0.140	0.053	0.011	0.068	0.004	0.004	0.002	0.000	0.141	0.282
Lower Bounds	−0.032	−0.045	−0.031	0.016	−0.004	−0.015	−0.005	−0.001	0.023	0.159
Upper Bounds	0.313	0.151	0.052	0.120	0.012	0.022	0.022	0.002	0.259	0.404

*Note.* EPD = Exposure to peer delinquency, DE = Depression, DRUG = Drug use. Lower and upper bounds are based on bias-corrected confidence intervals (99%).

## Data Availability

Not applicable.
